# The Immigrant Memory Collaborative: A Community–University Partnership to Assess African Immigrant Families’ Experiences with Dementia

**DOI:** 10.3390/ijerph19074075

**Published:** 2022-03-29

**Authors:** Manka Nkimbeng, Christina E. Rosebush, Kwame O. Akosah, Hawking Yam, Wynfred N. Russell, Gabriela Bustamante, Elizabeth A. Albers, Tetyana P. Shippee, Arundhathi P. Sasikumar, Joseph E. Gaugler

**Affiliations:** 1 Division of Health Policy and Management, University of Minnesota School of Public Health, Minneapolis, MN 55455, USA; rose0611@umn.edu (C.E.R.); akosa002@umn.edu (K.O.A.); ren00157@umn.edu (H.Y.); alber304@umn.edu (E.A.A.); tshippee@umn.edu (T.P.S.); gaug0015@umn.edu (J.E.G.); 2 African Career Education and Resources Inc., Brooklyn Park, MN 55445, USA; wrussell@acerinc.org (W.N.R.); asasikumar@acerinc.org (A.P.S.); 3 Program in Health Disparities Research, Department of Family Medicine & Community Health, University of Minnesota Medical School, Minneapolis, MN 55455, USA; busta027@umn.edu; 4 School of Public Health, Universidad San Francisco de Quito, Quito 170901, Ecuador

**Keywords:** Africans, immigrants, African immigrants, memory loss, dementia education, community-engaged research, community partnerships and needs assessment

## Abstract

Research suggests a disparity in the prevalence of dementia, with Black older adults having double the risk compared to their White counterparts. African immigrants are a fast-growing segment of the U.S. Black population, but the dementia care needs and resources of this population are not fully understood. In this paper, we describe the process of working collaboratively with a community partner and project advisory board to conduct a culturally informed project. Specifically, we describe the process of developing culturally informed instruments to collect data on dementia care needs and resources among African immigrants. Working together with a diverse project advisory board, a guide was developed and used to conduct community conversations about experiences with dementia/memory loss. Transcripts from six conversations with 24 total participants were transcribed and analyzed thematically by two independent coders in Nvivo. These qualitative findings were used to inform the development of a survey for quantitative data collection that is currently ongoing. Themes (e.g., cultural attitudes, challenges, and current resources) from the community conversations that informed the survey are described briefly. Despite the challenges of conducting research during a global pandemic, having trusting relationships with a partnering community organization and project advisory board facilitated the successful development of instruments to conduct preliminary dementia care research in an underserved population. We anticipate that survey results will inform interventions that increase education, outreach, and access to dementia care and caregiving resources for this population. It may serve as a model for community–university partnerships for similar public health efforts in dementia as well as other chronic disease contexts.

## 1. Introduction

One in three older adults in the United States (U.S.) will die from dementia [1]. The prevalence of dementia is projected to increase by 22% by 2025, which has led the U.S. Congress and the National Institute on Aging to prioritize dementia research and care [2]. Black older adults have double the risk of dementia but often receive diagnoses and treatment late, and have limited access to formal/paid services and supports (e.g., home care services and adult day care) [3,4,5]. These disparities arise from social and cultural factors such as discrimination, cultural values/norms, limited access to healthcare, and stigma [6,7,8,9]. Similar to their U.S.-born Black counterparts, African immigrants may also be at increased risk of poor dementia-related health outcomes.

There is a dearth of research on dementia in African immigrants, especially in the U.S. Current studies combine Afro-Caribbean and African immigrants, and have had mixed findings. Three earlier (1997, 2000 and 2001) studies in the United Kingdom (U.K.) showed that African and Afro-Caribbean immigrants had a higher prevalence of dementia, ranging from 8 to 34% [10,11,12,13], when compared to White persons. In a more recent (2015) study of 290 participants who received cognitive testing at a memory clinic in the U.K., Afro-Caribbean and African immigrants had lower scores on the Mini-Mental Status Exam and were diagnosed with dementia 4.5 years younger [14]. In the U.S., an analysis of the National Health and Aging Trends Study (NHATS) showed that Black immigrants (*n* = 125) were less likely to have dementia compared to U.S.-born Black older adults [15]. However, these results should be interpreted with caution because they relied on instruments that have not been validated in the African immigrant community [6], and thus may not represent the true cognitive health of this population.

As the current African immigrant population ages and migration to the U.S. increases, the older adult segment of this population will also grow. There are currently over 2 million African immigrants in the U.S.; almost one-fifth of them are over 55 years and 6.3% are over 65 years [16,17,18]. Indeed, the number of older adults gaining permanent residency in the U.S. in the past ten years has doubled [19,20]. Although African immigrants are healthier than their U.S.-born counterparts immediately after migration, a growing body of research suggests that immigrants’ health worsens with greater length of residence in the U.S. [21,22,23,24,25,26]. The growth of the older African immigrant population will likely result in a greater incidence of age-related conditions such as dementia.

In addition to the growing population of African immigrants, there is a growing burden of chronic diseases for older adults that may serve as risk factors for subsequent dementia or complicate the management of existing dementia among older African immigrants. Among adult African immigrants, the prevalence of hypertension ranged from 8.3 to 40% [27,28,29], diabetes from 34.6 to 65.5% [28,30,31], and obesity from 5.4 to 55% [29,32,33]. Thus, there is a need to understand dementia care burden (which often co-occurs with other chronic conditions) and related health outcomes for the growing African immigrant community.

Dementia research with communities such as African immigrants is limited because historically these communities have faced many social and cultural barriers to their participation and inclusion in research [34]. African immigrants reported that the following challenges limited participation in research: fear or stigma surrounding mental health or cancer [35,36,37], uncertainty/fear surrounding how data will be used [37,38,39], lack of time [39], and differential beliefs/perceptions about diseases [38]. Nevertheless, in a small sample (N = 34) of African immigrants, 91% reported a somewhat/very favorable attitude toward medical research and that medical research is important towards improving health outcomes [38].

Despite the growing population and incidence of chronic diseases in the African immigrants, there is a dearth of research on the dementia and care needs of this population. In this paper, we present a community-based participatory research project that utilized an exploratory mixed methods design [40] to identify dementia care needs and assets in the African immigrant community. Specifically, we describe the process of working with a project advisory board to create culturally informed instruments and using these to collect data in the community. This manuscript focuses on the procedures of this community–university partnership; related products and activities (e.g., interview guide, community conversation, surveys, etc.) are meant to be illustrative. The project was initiated prior to, but the majority of project activities occurred during, the COVID-19 pandemic. Subsequently, we also identify challenges encountered in the process of conducting community-engaged research during a global pandemic.

## 2. Methods

### 2.1. Community-Based Participatory Research Project Design

The need for a project exploring dementia care needs and resources in the African immigrant community was first identified by our community partner: African Career Education and Resources Inc. (ACER). ACER approached the University of Minnesota team based on past public health research and practice collaborations in the areas of nutrition and access to care. The new team (community and university) worked together to secure grant funding. The goals of the project were to: (1) develop a culturally informed community conversation guide that guides community conversations (focus groups) about dementia care and access, and (2) use the qualitative data to design and administer a dementia care needs and resources survey with the community.

Following grant funding, the community partner identified 20 community stakeholders with diverse experiences and expertise in community health, community organizing and development to form the project advisory board that meets quarterly. Principles, policies, and responsibilities of the project advisory board members were identified, discussed, and agreed upon by all at the November 2019 meeting. Project advisory members received a stipend (USD 25) for every meeting, and refreshments were provided. The meeting agendas were developed in collaboration with the advisory board members to match the project timeline and activities identified in the grant application.

The team prepared and submitted the project protocol for ethical review with the university’s Institutional Review Board (IRB) prior to initiation of all activities. This protocol was deemed ‘not human subject research’. All participant recruitment was led by the community partner and advisory board members during regular programming, and in-person activities occurred at locations that participants regularly attend. No identifying personal information was collected during data collection and oral consent was obtained prior to audio recordings.

### 2.2. Community Conversations Procedures

Community conversations served as phase one of this project and began with the creation of a guide consisting of 12 questions about current knowledge of dementia/memory loss, community needs, and current resources. The development of the community conversation guide was led by a committee of three volunteer project advisory board members with one university investigator. The committee members met outside of regular project advisory board meetings to develop the conversation guide iteratively (semi-structured questions; see Appendix A). A draft of the guide was presented to the full project advisory board for review and approval prior to use in the field. Following project advisory board members’ approval, the community partner identified locations for community conversations. Recruitment for community conversations was led by the community partner and project advisory board members. The community partner recruited participants during regular activities such as community education events, while project advisory board members spread the word within their networks and/or brought interested participants with them to the community conversations. When feasible, conversations were led by volunteer project advisory board members (3 of 6). Three community conversations were held in person with six participants at the first two and eight participants at the third.

Following the restriction of in-person activities due to the COVID-19 pandemic, the remainder of the conversations were conducted virtually via Zoom technology. Recruitment of participants for the virtual conversation was similar; the community partner identified potential participants and forwarded them to the research team for scheduling of the Zoom conversation. Community conversations were open to first (people born in Africa) and second-generation (people born in the U.S. to parents born outside) African immigrants residing in the community partner’s catchment area. However, attendance declined, with two participants attending one conversation, and two participants each who attended pre-arranged conversations alone were interviewed. Conversation durations ranged from 60 to 120 min. Community conversations were recorded with participants’ consent.

### 2.3. Qualitative Analysis

Audio recordings were transcribed by a professional transcription service, and data were analyzed using thematic content analysis as described by Braun and Clarke’s [41], six steps of qualitative analysis. In step 1, analyses began with each coder reading through all transcripts for familiarization and understanding of the context. In step 2, the transcripts were read for initial coding by coders using an inductive approach. Initial codes included “taking care of family in the home”, “not able to handle stimuli”, “study behavior on a day-to-day basis”, “being around them constantly”, “providers are not diverse”, “care in a facility is a last resort”, etc. In steps 3 and 4, the coders collated these initial codes and searched for preliminary themes, which were then reviewed and defined. The emerging themes and sub-themes were developed through an iterative process. For example, 18 overarching themes and 48 sub-themes identified initially were refined into 15 overarching themes. In step 5, themes were named and the codes within each emerging theme were then listed in a codebook that was used to code all community conversations in NVivo11. The coders did not identify any new codes/themes at this stage. Two team members coded each transcript, and preparation of this manuscript constitutes step 6 or reporting of study findings.

### 2.4. Trustworthiness and Rigor

Confirmability was achieved by having each transcript coded by two independent coders who met to discuss, define, and name the emerging themes. After consensus on final themes was achieved, they were presented to the project advisory board for feedback in preparation for the development of the survey about dementia care that is currently distributed in the quantitative phase of the project.

## 3. Results

Twenty-four African immigrants participated in the community conversations. The majority of the community conversation participants were women (67%), and 60% were over the age of 55 years. Participants were highly educated, with 65% having a Bachelor’s degree or higher, and 79% were born in Liberia or were children of Liberian immigrants. Liberian immigrants constitute a large fraction of the residents in the community partner’s catchment area. Amongst the 15 identified themes were descriptions of the following: cultural expectations around dementia care, the community’s dementia-related education needs, challenges or barriers to accessing healthcare, and attitudes about dementia and mental health. Additionally, there were themes related to resources available in the community to care for a person living with dementia and a comparison of dementia care available in the U.S. vs. African country of origin. Only the findings relevant to the survey development process are described here.

To describe how the relevant qualitative findings from the community conversations (Phase 1) were used to develop the quantitative survey (Phase 2), we briefly present a narrative of each theme and then provide a description of the unique questions or survey sections that captured this theme (see survey in Appendix B).

*Care situation:* Participants described their experiences of caring for a relative/friend living with memory loss or dementia. They described whether that relative lived with them in the U.S. as this participant noted “…*For instance, my mom is going through that memory loss and it is happening here*” (10th October conversation). Some of these relatives lived in their country of origin or were living in a U.S. facility (e.g., nursing home or assisted living).

In the survey, this theme was explored with four questions in the demographic section. It included questions such as “Are you currently caring for a family member or friend who is living with memory loss/dementia?”, “How long have you been caring for a person (or people) with memory loss/dementia”, and “Where does the person with memory loss/dementia live?”

*Current resources:* Participants described numerous places, organizations, and resources in the community where persons living with memory loss and dementia received education, social, emotional and financial support, transportation, and healthcare. For example, a participant said this about local professional associations: “*we have a nurses association, we have a group of grandmas and grandpas that we meet with occasionally and [provide] educational materials”* (10th October conversation) and another noted: “*for the Liberian community here in Minnesota we have these older folks program every Wednesday…they go and pick people up, they have discussions*” (6th October conversation*)*. They also described support groups and daycare programs as noted by this participant: “*I was told there is a daycare somewhere in Humboldt for elderly people*” (15th October conversation).

Resources were also captured in the background section of the survey with one main question—“*If you needed it for yourself or a family member/friend, where would you seek support for memory loss/dementia?*”—with thirteen sub-sections to identify the specific type of organization (i.e., religious, healthcare provider, country of origin, etc.) available to help.

*Dementia knowledge:* In all the conversations, participants identified some signs and symptoms of dementia, such as “*All of a sudden we started getting the feeling that she [mom] was forgetting stuff*” (15th October conversation) or “*Dementia has stages I believe, the stages kind of determine how bad the situation is*” (15th October conversation). There was very limited discussion about diagnosis and treatment of dementia but there was overwhelming agreement that community dementia education was needed.

“*I think the early education is important and crucial, early detection can be helped really well… But if we are not aware and have a dementia patient, we will not be able to identify it and call for help from professionals. And not too many Africans here do doctor things like check-ups*” (15th October conversation).

Dementia knowledge in the quantitative survey was captured with the validated Dementia Knowledge Assessment Scale (DKAS) [42,43] that included questions from the following domains: causes and characteristics, communication and behavior, care considerations and risk, and health promotion in the context of dementia.

*Barriers to accessing care:* Conversation participants described many barriers and challenges to accessing care. These included immigration status as this participant stated, “*Most of the time, our loved ones that come will not be part of the system right. If your immigration status was questionable, how would you reach out for help? So those types of things and sometimes you won’t be eligible for support or aid*” (10th October conversation). Other challenges included language/accent barriers, lack of diversity in healthcare providers, fear to seek medical care-related to racism and lack of trust in the healthcare system, limited finances, multiple caregiving responsibilities, etc.

In the survey, these barriers and challenges were explored using a Likert-type scale that incorporated this prompt: “*Indicate your level of agreement with each statement*”. The statements were the various challenges identified in the conversations and included: “*Fear and mistrust of western medicine is a challenge to memory loss/dementia care*” or “*Members of my family are unable to seek care because of their immigration status*” or “*Limited language or access to translation is a barrier to seeking memory loss/dementia care*”. Response options ranged from strongly disagree, disagree, neutral, agree, and strongly agree. Additionally, the validated Everyday Discrimination Scale [44,45] was used to further elucidate the experience of discrimination.

*Attitudes toward dementia and mental health:* Finally, participants’ attitudes and beliefs surrounding dementia care, caregiving, and mental health were very evident from community conversations. Participants described a preference for care from family members in the home as this participant stated: “*The culture, meaning the culture that we take care of our own no matter how*” (10th March conversation). They also described that there was stigma around dementia in the community—“*Stigma—[people] don’t want memory loss on their chart because people treat you differently with diagnoses*” (14th December conversation)—and seeking care in a U.S. facility was a last resort.

This content was also explored in the survey using a Likert-type scale as described above. It included statements of various attitudes and beliefs, such as: “*Families are responsible for caring for people with memory loss/dementia in the family*”, or “*Caring for someone with memory loss/dementia in my family happens at home*”, or “*African immigrants are very secretive about memory loss/dementia*”. Response options ranged from strongly disagree, disagree, neutral, agree, and strongly agree.

*Survey Development Process:* The university team drafted survey questions and then shared them with the project advisory board for review. Members were invited to edit and add to the survey using track changes in Microsoft Word. Following this, the university team revised the survey to incorporate all project advisory board members’ feedback. A revised version was then brought to a project advisory board meeting for discussion. A final copy of the survey was created that incorporated all of the changes suggested during the meeting prior to implementation (See Appendix B). Survey data collection began in June 2021 and is currently ongoing (over 170 surveys have been completed so far). The community partner identified other community-based organizations (e.g., churches, mosques, etc.) for community data collection. The community partner approached the leader and coordinated a date and time for in-person data collection at these community-based locations and events. Following this, the team then attended these events to collect in-person surveys, which were completed by hand using paper and pen or virtually on tablets. Due to the global pandemic, a virtual survey link was also developed and distributed through community networks (e.g., community partner, project and project advisory board members’ social media accounts).

## 4. Discussion

In this manuscript, we described the process of collaboration between a university and community partner to develop culturally informed instruments (i.e., a community conversation guide and subsequent survey) to collect data about dementia care needs and resources among African immigrants in the U.S. Community conversations (phase 1) with participants revealed five themes that warranted further exploration in the phase 2 community survey: (1) current care situation (i.e., whether people were currently caring for someone with dementia and where that person resided), (2) resources available to support the family, (3) current knowledge about dementia, (4) barriers to accessing dementia care, and (5) attitudes towards dementia care.

Although there are many reasons to account for the dearth of research with African immigrants, there is an urgent need for research with this community. Limited research participation of underserved communities affects the generalizability of study results and ultimately decisions related to best practices and clinical outcomes [34,46]. Additionally, in accordance with the tenets of health equity and distributive justice, there is a need to ensure that research is representative of all communities to ensure equitable distribution of risks and benefits [47]. Despite current and historical challenges, engaging community gate keepers, addressing religious and immigration factors, maximizing the research team’s cultural competence and promoting altruism through health education are some successful strategies for outreach and recruitment of African immigrants into research [48,49]. This manuscript relates one successful approach of collaborating with a community partner and project advisory board to conduct a culturally informed project with African immigrants.

Having limited information/data on dementia in the African immigrant community hinders health care providers’ ability to provide timely diagnoses and culturally congruent care. With the growing diversity of the U.S. older adult population, it is essential to understand disease trends and risk factors as well as outcomes of treatments and interventions for these populations [34,50]. Currently, forty percent of persons with dementia in the U.S. are undiagnosed [51]. Studies from Europe showed that a limited understanding of cultural taboos, stigmas, and expectations of family caregivers affects the delivery of dementia care [52,53]. Data from this project can lead to a greater understanding of cultural perspectives around dementia and family caregiving, which can inform improved dementia care for immigrant communities.

In addition to a limited understanding of dementia care burden in African immigrants, few efforts exist to culturally adapt dementia care and caregiving interventions for racial/ethnic minority and underserved populations [54,55,56,57,58]. Moreover, these efforts are not systematically documented and none of the current adaptations focus on African immigrants [57]. Interventions for other immigrant groups include: Our Family Journey for Vietnamese American caregivers, psychoeducational skill training for Chinese American caregivers, and Webnovela Mirela for Hispanic caregivers [55]. Education delivered within the community by a trusted source (e.g., a member of the community) has the potential to improve health-related quality of life and social well-being for family caregivers [59]. Research shows that culturally tailored interventions are more effective than generic health interventions for various disease contexts [58,60,61]. Foundational research with African immigrants, such as this study, is necessary to understand the burden of dementia and also inform cultural tailoring of future dementia interventions for this growing population.

Conducting community-engaged research in times of a global pandemic presented challenges that extend beyond research conducted in pre-pandemic times. A major challenge of this project was the inability to safely convene in-person activities. In-person community conversations and project advisory board meetings were transitioned to virtual events using web conferencing technology. Attendance at these virtual events decreased (compared to in-person events), but it is unclear if this was related to the change in modality or the pandemic in general. Secondly, recruitment and enrollment of older adults for community conversations became difficult virtually because of challenges accessing technology. Third, the COVID-19 pandemic presented unique mental health circumstances and scheduling challenges for many on the research team and in the community. Flexibility in scheduling and processing space was offered as needed during virtual PAB meetings. Following the development of the quantitative survey, the data collection plan was revised to include virtual approaches due to the challenge of convening in-person data collection events at community-based organizations.

This project and its findings are not without limitations. Although we attempted to recruit participants from more African countries, the majority of the sample were Liberian and community conversations conducted in one U.S. state might not reflect the experiences of all African immigrants in the U.S. Secondly, due to the pandemic, the number of participants and community conversations were fewer than anticipated. However, data saturation (where no new additional themes emerged) was achieved after the third community conversation. Despite these limitations, this community-engaged project provides foundational data for understanding dementia care experiences of African immigrants. Additionally, forthcoming quantitative results will provide information on the barriers, challenges and resources available for dementia care and caregiving for African immigrants.

## 5. Conclusions

To the best of our knowledge, this is the first study to explore dementia assets, challenges, and care practices with African immigrants in the U.S. Through a community–university partnership, we developed culturally informed instruments to collect data about dementia care needs and resources among African immigrants. Community conversations with participants reveal five themes that are currently being explored in the quantitative phase of the project. Utilizing a community-engaged approach through a community–university partnership is one approach to successfully implementing a culturally informed research project with African immigrants. Understanding the dementia disease and care burden of African immigrants will subsequently inform adequate tailoring of dementia education, resources, and interventions.

## Data Availability

Due to privacy issues, the authors are unable to share the qualitative data for this study.

## Appendix A. African Immigrant Memory Loss Project Community Conversation Guide

**Phase 1: Current Knowledge about Memory loss**

1. Please tell us when you first heard about memory loss/dementia?

*Probes*

a *Was it in your country of origin, the U.S. or somewhere else?*
b *How did you hear about it, video, your doctor or some other means?*
c *Do you know anyone in the African immigrant community with memory loss? If yes, tell us more.*

2. In your opinion, what is memory loss or dementia?

*Probes*

a *Describe the signs and symptoms of memory loss/dementia.*
b *How is memory loss/dementia diagnosed?*

3. In your opinion how is memory loss /dementia currently managed?

*Probes*

a *Is there a treatment or cure for memory loss/dementia? If yes, what is it?*

4. Describe how you, your family and others in the African immigrant community care for people with memory loss.

*Probes*

a. *Do you/your family member(s) have a primary care provider?*
b. *Do you/your family see a doctor as often as you need to?*

5. Have you/your family discussed what will happen if you develop memory loss?

*Probes*

a. *Tell us where you would go to get medical care and resources for a family member with memory loss.*
b. *Do you know what an Advanced Directive is? Do you have one, have you discussed this with your family? (Moderator- please describe AD and healthcare agent, if needed).*
c. *Who is aware of your healthcare wishes i.e., who will make decisions for you when/if you are not able to decide for yourself?*

**Phase 2: Community Needs**

6. Do you think your community needs any education about memory loss/dementia?

*Probes*

a. *Does the community need education on signs and symptoms of memory loss/dementia?*
b. *Do they need more information about treatment and management of memory loss?*
c. *Should there be education on resources available in the county, state of MN, and country?*

7. Are there any challenges to caring for people with memory loss in your community?

*Probes*

a. *What are some challenges that African immigrants with memory loss and those caring for them might face?*
b. *Are there barriers (language, culture, income, discrimination etc.) that can impact African immigrant’s ability to get care or services?*
c. *What are some reasons that someone might forgo seeking treatment for memory loss?*
d. *Are you aware of anyone in the community who is not able to get healthcare because of their immigration/visa status*
e. *Are there ways in which your life has changed since coming to the U.S that can impact the care of family members with memory loss?*

8. Some people decide to seek care for family members with memory loss at specialized programs and services such as adult day care, assisted living or nursing homes. Would you or your family consider this type of care?

*Probes*:

a. *Have you or any member of your family received care from any of these facilities?*
b. *Would you consider this type of care facility for yourself or a family member? Yes/No and why?*
c. *Do you think people in the African immigrant community would consider these types of facilities?*

9. Do you or other family members plan to retire back in your country of origin or somewhere else? How would things change if you or a family member were to have memory loss?

*Probes*

a. *Are all your family members in the U.S? Does that impact the care for a person with memory loss in your family?*
b. *Who is the primary caregiver for older adults in your family? Are they in the U.S. currently?*

**Phase 3: Current Resources**

10. Culture affects how we do things: Tell us if there are unique resources in the community that can benefit African immigrants and their families?

*Probes*

a. *Does your place of worship or African cultural group(s) have resources that can help you or your family manage life in the U.S.*
b. *Are there any things/places or resources from back home (or somewhere else) that you think can help people with memory loss here?*
c. *Are you aware of any herbals, oils or traditional medicine that people in the African immigrant community use for memory loss?*

11. Do you feel you have more resources here (in the U.S) or back home to care for a family member with memory loss?

*Probes*

a. *How so?*

12. Are there resources/strengths in the African immigrant community that can facilitate caring for a person with memory loss?

*Probes*

a. *Are there any unique African practices or things that can help those with memory loss or people caring for someone with memory loss?*

## Appendix B. The Immigrant Memory Collaborative Survey

**Section 1: About you**

1. Which of the following describes your gender?
  - Male
  - Female
  - Other (specify): ______________
2. How old are you? ______________
3. What is your zip code? ______________
4. Where were you born?
  - I was born in Africa
  - I was born in the United States (U.S.) and my parents were born in Africa
  - Other (specify): ________________
5. What country in Africa are you (or your parents) from? _________________
6. What year did you first come to the U.S. to live?
  - Year: ______________
  - I was born in the U.S.
7. What is the primary language you speak at home? ______________
8. What other languages, if any, do you speak at home? ______________
9. What is your marital status?
  - Single/Never married
  - Married
  - Separated/Divorced
  - Widowed
  - Cohabitating
10. What is the highest level of education you have completed?
  - Less than high school
  - High school graduate/GED or equivalency
  - Associate degree/Technical college/Training
  - Bachelor’s degree
  - Master’s degree
  - Doctorate (for example: MD, JD, DDS, PhD)
11. What is your employment status?
  - Working a full-time job
  - Working a part-time job
  - Self-employed
  - Not employed for pay (for example: homemaker or stay-at-home parent)
  - Retired
  - Unable to work due to ill health
  - Currently seeking employment
12. What is your total yearly household income?
  - Less than $39,999
  - $40,000 to $79,999
  - $80,000 to $119,999
  - $120,000 or above
13. Do you have a person you regularly see for your health care needs (also known as a primary care provider)?
  - Yes
  - No
14. Are you a U.S. citizen or permanent resident? We are asking because it can affect access to health services. **This information will not be shared with anyone.**
  a. Yes
  b. No
  c. Prefer not to answer
15. Why did you (or your parents) first come to the U.S.? We are asking because it can affect access to health services. **This information will not be shared with anyone.**
  a. Education
  b. Economic hardship
  c. To join family/Marriage
  d. Asylum/Refugee
  e. Job opportunities
  f. Other (specify): ______________
16. Do you have health insurance?
  - Yes
  - No **[skip ahead to question 18]**
17. What type of health insurance do you have?
  - Private health insurance
  - Government subsidized/sponsored health insurance (for example: Medicare, Medicaid, Affordable Care Act/Obamacare, Military)
  - Don’t know
18. How do you get to health care appointments? We are asking because transportation can be a barrier to receiving health care.
  - Private car
  - Family car
  - Public transportation (for example: bus or train)
  - Paid transportation service (for example: Uber or Lyft)
  - Other (specify): ______________
19. Are you currently caring for a family member or friend who is living with memory loss/dementia?
  - Yes
  - No **[skip ahead to question 24]**
20. How long have you been caring for a person (or people) with memory loss/dementia?
  - Less than 6 months
  - 6 months–1 year
  - 1–3 years
  - 3–5 years
  - 5–10 years
  - 10 years and above

**If you provide care for multiple people with memory loss/dementia, please think about the person you provide *the most care* for while answering questions 21–23.**

21. What is your relationship to the person with memory loss/dementia?
  - I am their spouse or partner
  - I am their child
  - I am their grandchild
  - I am their niece or nephew
  - I am their sibling (brother or sister)
  - Other (specify): ______________
22. Where does the person with memory loss/dementia live?
  - In the U.S.
  - In Africa (for example: you are providing money to help with their memory/dementia care, coordinating healthcare services, training home healthcare workers, etc.)
  - Somewhere else (specify): ______________
23. If a doctor has diagnosed the person with memory loss/dementia, what is the specific diagnosis (type of dementia)?
  - Alzheimer’s disease
  - Vascular dementia
  - Lewy Body dementia
  - Frontotemporal dementia
  - Mixed dementia
  - A doctor has provided a diagnosis, but I am unsure what it is
  - A doctor has not provided a diagnosis
  - Other, please specify:______________
24. If you needed it for yourself or a family member/friend, where would you seek support for memory loss/dementia? By support, we are referring to dementia education, healthcare, transportation, or other services. ***Please select all that apply and provide the specific name of the organization.***
  - My religious organization (for example: church or mosque): ______________
  - My doctor, clinic, or community health center (for example: Hennepin Healthcare, Fairview, etc.): ______________
  - My local pharmacy: ______________
  - My friends, neighbors, or other community members
  - My state or county agency (for example: county educational events or public health nursing services): ______________
  - My country-of-origin embassy or consulate: ______________
  - My African tribal/village/cultural group organization: ______________
  - My African country of origin organization: ______________
  - Another African organization: ______________
  - A professional association (for example: Nigerian Nurses Association): ______________
  - An African adult day care center: ______________
  - A senior building or residential program: ______________
  - Another not-for-profit, business, or organization: ______________

**Section 2: Dementia knowledge and beliefs**

25. The following statements are common beliefs about memory loss/dementia. Some are true and some are false. We are asking so that we can prepare educational materials that meet the needs of our community. ***For each statement, check one box (True or False). It is okay if your answers are not correct.***

**To the Best of Your Ability Indicate Whether These Statements Are True or False.**	**True**	**False**
a. Most forms of dementia do not generally shorten a person’s life		
b. Blood vessel disease (vascular dementia) is the most common form of dementia		
c. People can recover from the most common forms of dementia		
d. Dementia is a normal part of the aging process		
e. Dementia does not result from physical changes in the brain		
f. Planning for end-of-life care is generally not necessary following a diagnosis of dementia		
g. Alzheimer’s disease is the most common form of dementia		
h. It is impossible to communicate with a person who has advanced dementia		
i. A person experiencing advanced dementia will not generally respond to changes in their physical environment		
j. It is important to correct a person with dementia when they are confused		
k. People experiencing advanced dementia often communicate through body language		
l. Uncharacteristic behaviors in a person experiencing dementia are generally a response to unmet needs		
m. Medications are the most effective way of treating behavioral symptoms of dementia		
o. People experiencing dementia do not generally have problems making decisions		
p. Movement is generally affected in the later stages of dementia		
q. Difficulty eating and drinking generally occurs in the later stages of dementia		
r. People with advanced dementia may have difficulty speaking		
s. People experiencing dementia often have difficulty learning new skills		
t. Daily care for a person with advanced dementia is effective when it focuses on providing comfort		
u. Having high blood pressure increases a person’s risk of developing dementia		
v. Maintaining a healthy lifestyle does not reduce the risk of developing the most common forms of dementia		
w. Symptoms of depression can be mistaken for symptoms of dementia		
x. The sudden onset of cognitive problems is characteristic of common forms of dementia		
y. Exercise is generally beneficial for people experiencing dementia		
z. Early diagnosis of dementia does not generally improve quality of life for people experiencing the condition		

26. The following statements are about behaviors and attitudes about memory loss/dementia in our community. There are no right or wrong answers. We are asking so that we can better understand what people think about memory loss/dementia. ***For each statement, check one box (Strongly disagree, Disagree, Neutral, Agree, or Strongly agree).***

**Indicate Your Level of Agreement with Each Statement.**	**Strongly Disagree**	**Disagree**	**Neutral**	**Agree**	**Strongly Agree**
a. It is difficult to return to Africa after living in the U.S. for a long time					
b. Caring for someone with memory loss/dementia in my family happens at home					
c. Families are responsible for caring for people with memory loss/dementia in the family					
d. Memory/loss dementia is more common among African immigrants living in the U.S. than it is among people in Africa (due to lack of social opportunities, dependence on others, less meaningful roles for elders, etc.)					
e. African immigrants are very secretive about memory loss/dementia					
f. Caregiving for memory loss/dementia is not a big deal for African immigrants in the U.S. We’re always there to care for our family members					
g. African immigrants prefer to avoid bad news					
h. There is limited awareness about mental health in African immigrant community					
i. African immigrants do not like to talk about death or dying					
j. I first learned about memory loss/dementia in Africa					
k. I first learned about memory loss/dementia in a healthcare job					
l. Moving a family member with memory loss/dementia to a facility (for example: nursing home or assisted living) is a last resort					
m. I’ve heard a lot of bad things about caring for a person with memory loss/dementia in U.S. facilities					
n. People with memory loss/dementia are crazy or have been bewitched					
o. Caring for a person with memory loss/dementia in Africa is easier because other family members and the community are there to help					

27. The following statements are about types of discrimination you may have experienced. We are asking because discrimination is linked to health care and health outcomes. ***For each statement, check one box (Never, Rarely, Sometimes, Often)***.

**In Your Day-To-Day Life, How Often Do The Following Things Happen to You?**	**Never**	**Rarely**	**Sometimes**	**Often**
a. You are treated with less courtesy than other people are.				
b. You are treated with less respect than other people are.				
c. You receive poorer service than other people at restaurants or stores.				
d. People act as if they think you are not smart.				
e. People act as if they are afraid of you.				
f. People act as if they think you are dishonest.				
g. People act as if they’re better than you are.				
h. You are called names or insulted.				
i. You are threatened or harassed.				

28. The following are some challenges that people may experience when they seek care for memory loss/dementia in the U.S. There are no right or wrong answers. We are asking so that we can identify services that may help people overcome challenges to care. ***For each statement, check one box (Strongly disagree, Disagree, Neutral, Agree, or Strongly agree).***

**Indicate Your Level of Agreement with Each Statement.**	**Strongly Disagree**	**Disagree**	**Neutral**	**Agree**	**Strongly Agree**
a. Cultural issues limit people from getting care for memory loss/dementia					
b. Fear and mistrust of western medicine is a challenge to memory loss/dementia care					
c. Racism in medical care is a challenge to seeking memory loss/dementia care					
d. Members of my family are unable to seek care because of their immigration status					
e. Limited income and finances is a challenge to seeking memory loss/dementia care in facilities such as assisted living and nursing homes					
f. Limited income/finances is a challenge to bringing in care providers (for example: nurse, home health aide) to provide memory loss/dementia care at home					
g. Limited knowledge about memory loss/dementia is a challenge to accessing medical care					
h. Members of my family are unable to seek memory loss/dementia care because they don’t have insurance					
i. Members of my family are unable to seek memory loss/dementia care because they don’t have transportation					
j. Limited language or access to translation is a barrier to seeking memory loss/dementia care					
k. Having multiple responsibilities (for example: parent, school, work) is a challenge to caring for someone with memory loss/dementia					
l. Limited diversity in medical providers (for example: doctors and nurses) is a challenge to seeking memory loss/dementia care					

**
  Section 3: Caregiver experiences
  **

29. If you are not currently caring for a family member or friend who is living with memory loss/dementia, *skip ahead to question 31*. The following statements are about your experiences as a caregiver. We are asking to better understand what caregivers in our community need. *For each statement, check one box (Never, Rarely, Sometimes, Often).*

**Check the Response That Best Describes How You Feel.**	**Never**	**Rarely**	**Sometimes**	**Often**
a. Do you feel that because of the time you spend with your care recipient that you don’t have enough time for yourself?				
b. Do you feel stressed between caring for your care recipient and trying to meet other responsibilities for your family or work?				
c. Do you feel strained when you are around your care recipient?				
d. Do you feel uncertain about what to do about your care recipient?				

30. The following statements are about how confident you are finding services and supports to meet the needs of your care recipient. We are asking so that we can help connect caregivers with available services and supports. ***For each statement, check one box (Very unconfident, Unconfident, Neutral, Confident, Very confident).***

**Indicate How Confident You Are Right Now That You Can.**	**Very Unconfident**	**Unconfident**	**Neutral**	**Confident**	**Very Confident**
a. Handle any problems your care recipient has					
b. Handle any problems that might come up in the future with your care recipient’s care					
c. Do something to keep your care recipient as independent as possible					
d. Get answers to all your questions about your care recipient’s problems					
e. Find organizations or agencies in the community that provide services to help your care recipient					
f. Get answers to all of your questions about these services					
g. Arrange for these services yourself					
h. Find ways to pay for these services					

**
  Section 4: Open-Ended Questions
  **

31. What are our community’s major strengths and weaknesses for addressing the needs of people living with memory loss/dementia and their families?
32. If you are currently providing care to a family member or friend living with memory loss/dementia, what type of care do you provide to them (for example: help with eating, bathing/dressing, finances, transportation)? Are there parts of caregiving that have been particularly easy or difficult for you? If so, what are they?
33. In your opinion, what is the ideal caregiving scenario for someone living with memory loss/dementia in our community (community setting with family vs. nursing home/institutional care)? In your experience, is the ideal caregiving scenario possible? Why or why not?
34. If you are a caregiver for someone with memory loss/dementia ***in your country of origin***, please describe the type of care you provide (for example: providing money to help with their memory/dementia care, coordinating healthcare services, training home healthcare workers, etc.). We are interested in learning more about long-distance caregiving.
